# The effect of imposing a higher, uniform tobacco tax in Vietnam

**DOI:** 10.1186/1478-4505-4-6

**Published:** 2006-06-26

**Authors:** Hoang Van Kinh, Hana Ross, David Levy, Nguyen Thac Minh, Vu Thi Bich Ngoc

**Affiliations:** 1 Ph.D candidate, MSc, Vietnam Commercial University, Vietnam; 2 Senior Scientist, Research Triangle Institute, USA; 3 Professor, University of Baltimore, USA; 4 Instructor, Vietnam Commercial University, Vietnam; 5 Econometrician and instructor, Institute of Finance, Vietnam

## Background

Tobacco use increases the risk of many fatal diseases such as cancer, emphysema, heart disease and other circulatory diseases[1]. If trends in tobacco use continue, approximately 500 million people alive today will die from smoking, and, by 2030, tobacco is expected to be the leading cause of premature death, accounting for about 10 million deaths per year.[2]

While smoking prevalence has been decreasing in many of the more developed nations, rates have been high and increasing in many of the poorer nations. For example, smoking rates among Vietnamese males 15 year of age and older was 50% in 1997–98,[3] but rose to 56% in 2002.[4]

The Government of Vietnam is aware of the impact of smoking on public health. It has launched a campaign against smoking by issuance of a Government Resolution on National Tobacco Control Policy[5] in 2000 with target of reducing the tobacco use prevalence rate to 20% for males and 2% for females in 2010. Vietnam was one of the first Asian nations to sign the World Health Organization's Framework Convention for Tobacco Control in 2003.

Tobacco tax increase is among the measures suggested in the 2000 Resolution to achieve its goal of lower smoking prevalence. Other components of the tobacco control program include total ban on cigarette advertising, ban on distributing free cigarette samples, ban on vending machines selling cigarettes, and ban on smoking in all public places. Research evidence shows that imposing taxes on tobacco, as part of a comprehensive tobacco control program, is among the most effective methods of reducing tobacco use.[6,7] Higher taxes create incentives for some regular smokers to quit smoking, help prevent the young from initiating smoking, and reduces consumption among continuing smokers.

The majority of evidence on the price responsiveness of tobacco demand is for high-income countries were data and the research capacity exists. Estimates of the price-elasticity for overall cigarette demand fall in a relatively wide range due to model specification, data issues and estimating methodology,[8,9] but the majority center in the relatively narrow range from -0.25[10] to -0.5.[11,12] Evidence does indicate that low income groups in the high income countries are more sensitive to cigarette prices compared to higher income groups.[13]

Recent studies have begun to focus on tobacco consumption in low-income countries, including South East Asia. For Indonesia, Djutaharta et al. (2002)[14] used time-series data to estimate cigarette price elasticities in Indonesia ranging from -0.32 to -0.57, Adioetomo et al. (2001)[15] used household level survey data to obtain a conditional (i.e., on quantity smoked per smoker) price elasticity of -0.6, but the impact of price on smoking participation (i.e., the decision to smoke) was not significant. For Sri Lanka, Arunatilake (2002)[16] used household level data and estimated that the price elasticity was -0.53 for the whole sample and between -0.68 and -0.29 for the poorest two quintiles, Arunatilake and Opatha (2003) [17]used aggregate monthly data and estimated price elasticities ranging from -0.227 to -0.908. A price elasticity of tobacco demand in Thailand of -0.67 was estimated by Supakorn (1993)[18] using aggregate tobacco consumption. Isra et al. (2003) [19] used a linear expenditure system and household level data, and found the price elasticity of the demand for tobacco products of -0.39. They also found that poorer smokers were more responsive to tobacco prices than their richer counterparts. Karki et al. (2003)[20] estimated a conditional price elasticity of cigarette demand of -0.42 and a total price elasticity of -0.88 in Nepal using household data. For Myanmar, Nyo Nyo et al. (2003)[21] obtained a total price elasticity of -1.62 using household data. A WHO study[22] using time series data obtained an overall price elasticity in Vietnam of -0.53 for cigarettes, but did not take into account a possible substitution into other tobacco products. Also for Vietnam, Laxminarayan and Deolalikar (2004),[23] controlling for use of other tobacco products, obtained a price elasticity of smoking initiation of -1.18, but did not find a significant impact of cigarette price on quitting. Guindon, et al. (2003)[24] estimated the demand for cigarettes in South-East Asia using panel data. They obtained short-run price elasticity estimates ranging from -0.17 to -0.78, with most estimates clustering at around -0.74, and long-run elasticities ranging from -0.4 to -1.21. Few studies suggest a limited impact of price on smoking behavior, but a study of cigarette demand in China and Russia[25] obtained elasticities ranging from 0 to -0.15 using micro-level data. Although variations exist in the elasticity estimates and in the quality of the studies, the evidence strongly confirms a negative relationship between smoking and cigarette prices.

Public policy makers can use tobacco taxes to manipulate cigarette price. Tobacco tax rates vary from country to country. In high-income countries, the tax component often accounts for at least two-thirds of the retail price of a pack of cigarettes. In low-income countries, on the other hand, it generally accounts for less than half of the retail price.[26] In the countries attempting to reduce tobacco consumption, the tax component is typically between two-thirds and three-fourths of the retail price of a pack of cigarettes.[6]

**Figure 1:**
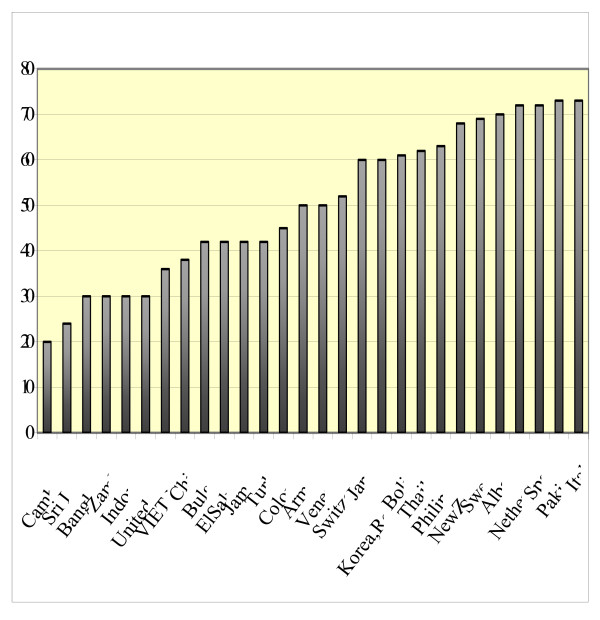
**Tax rate as percentage of price of Vietnam and selected countries**. *Source: *Frank J. Chaloupka, Teh-wei Hu, Kenneth E. Warner, Rowena Jacobs, and Ayda Yurekli, 2000.

Vietnam has relatively low cigarette taxes leading to low cigarette prices.[27] (see Figure 1). Cigarette prices relative to income are higher than many other countries, but the price-income relationship has fallen quite dramatically in Vietnam between 1990 and 2001.[28]

The effect of tobacco taxes may depend not only on the size, but also on the form of the tax. Specific tobacco taxes are added as a fixed amount to the price of cigarettes, while ad valorem taxes, such as value-added taxes or sales taxes, are a percentage of the base price. Ad valorem taxes may be imposed at the point of sale or, as in China, Vietnam and many African countries, on the wholesale price so that the retail price already includes the tax.[29] Taxes may also vary according to the origin of the manufacturer or the type of product. For example, some governments impose higher taxes on cigarettes produced abroad than on domestically produced ones, or by type of cigarette such as non-filtered or filtered cigarettes. Among the 114 countries with available information in the Tobacco Control Country Profiles (WHO, 2000),[30] 95 levy a uniform tax on tobacco, while 19 levy different tobacco tax rates based on types of products and sources of materials, 10 of which were part of the former Soviet Union and or the Soviet bloc. Vietnam is also one of the countries imposing differential ad valorem tax rates.

Prior to 1999, Vietnam imposed a tax rate of 70% on filtered cigarettes produced mainly from imported materials, of 52% on filtered cigarettes produced mainly from domestic materials, and a tariff rate of 70% on imported cigars. Since 1999, Vietnam has imposed separate tax rates on three types of cigarettes: a tax of 65% is imposed on filtered cigarettes made from imported materials; a tax of 45% is imposed on filtered cigarettes made from domestic materials; and a tax of 25% is imposed on non-filtered cigarettes made from domestic materials.[31] Non-filtered cigarettes produced from domestic materials, besides having low production costs, are taxed least, and are thus sold at even lower prices relative to filtered cigarettes. Thus, the current three-tiered tax structure exacerbates price differences, with the price of many domestically produced cigarettes around US$0.07 per pack compared to a price of foreign brand cigarettes such as 555 and Marlboro that can reach up to US$1[32].

Although higher cigarette taxes can reduce smoking and the associated health problems, they have implications for the spending pattern among smokers and for tax revenue. Since low price cigarettes are more likely to be consumed by those with low incomes, the low price of unfiltered cigarettes potentially creates more incentive for those with low income (including youth) to smoke, those that can least afford it. Raising taxes on low priced cigarettes will raise price, which could increase that burden. However, if the low-price smokers are sufficiently responsive to price, they may actually reduce consumption through quitting or cutting back on quantity smoked, and have a lower financial burden. In that case, government revenue from cigarette taxes could actually fall.

This paper considers how smoking rates among different income classes is affected by the current three-tiered tax structure, and the potential effect on burdens of the poor, and then considers the effect of raising taxes on low priced cigarettes. Specifically, this paper examines how the multi-level tobacco tax policy affects low-price cigarette smokers, estimates the price elasticity of demand for cigarettes by income groups, and finally analyzes the effect of imposing a single uniform tax at the level of 65% on consumption and government revenue.

## Methods

### Description of the data

Due to the lack of a sufficiently detailed time series data, cross sectional data is employed to examine smoking behavior among various income groups and the financial burden that it imposes, and to estimate smoking elasticities of those that consume primarily the low price cigarettes. We use the second Vietnam Living Standard Survey (VLSS) which was conducted by the General Statistical Office from December 1997 to December 1998 with a sample size of 6000 households with 28,518 individuals. Quality control was implemented in several stages, from questionnaire design, data entry, random unexpected field visits, and consistency checks after the survey was completed.

The sample was selected using a three staged random stratified cluster sampling. Using sampling weights, the data yield unbiased population estimates at the national level and are disaggregated by urban and rural residence and the seven major regions. Adjustments for clustering and stratification were made to yield more accurate standard errors for hypothesis testing using the STATA statistical software.

The VLSS dataset contains variables on a wide range of socio-economic factors including education, employment, income, family structure, location, and living standards. The questionnaire has a separate section on smoking for people age 6 and older. Individual are first asked if they had ever smoked cigarettes for a period of 6 months or more. Those who responded in the affirmative were asked whether they currently smoke, how many cigarettes they smoke each day, and the amount of money spent on cigarettes over the past 12 months. A cigarette smoker is person who is at least 15 year or age, currently smokes cigarettes and has smoked at least for six months. It is assumed that all current smokers are daily smokers. The survey also asked about expenditures on pipe smoking. The answer to this question was used to identify a pipe smoker. There are respondents that smoke both cigarettes and pipe tobacco.

Besides the household questionnaire, a separate questionnaire collects information related to characteristics at the level of the commune, the lowest administrative unit in Vietnam with an average population of 7000. Three representative retail outlet owners/workers (who are familiar with commune prices) were interviewed about prices of goods and services in the commune. Information on two types of cigarettes (a high priced filtered cigarette known as 555 and a lower price filtered cigarette known as VINATABA) was obtained, yielding 6 price data points from each commune. This information was used to calculate an average commune price.

### Analysis of the users of low price cigarettes

We first consider the percentage of smokers that smoke the different types of cigarettes. Because information is unavailable to specifically distinguish between types, we classify smokers by price of cigarettes. The average prices paid for each of the types is estimated using expenditure data from the VLSS. Average prices paid per pack were calculated by dividing tobacco expenditures by the number of cigarettes smoked and multiplying by 20.

In Vietnam, cigarette retail prices vary substantially across types and also vary within type. Based on our investigation of prices in the Hanoi market in June 2003, non-filtered cigarette prices are usually between 1000 and 2000 VND^1 ^per pack (between $0.07 and $0.13 at an exchange rate of 15,000 VND per $1 USD in 2003). Prices of 555 or smuggled Marlboros are among the highest and range between 12,000 and 15,000 VND per pack (between $0.80 and $1.00). A low price is defined as less than or equal to 1/3 of the highest cigarette retail price, based on actual prices and expenditure patterns observed from the data. Since the highest retail price in 1998 was 15,000 VND, the low price is considered 5,000 VND ($0.33) and below.

**Figure 2:**
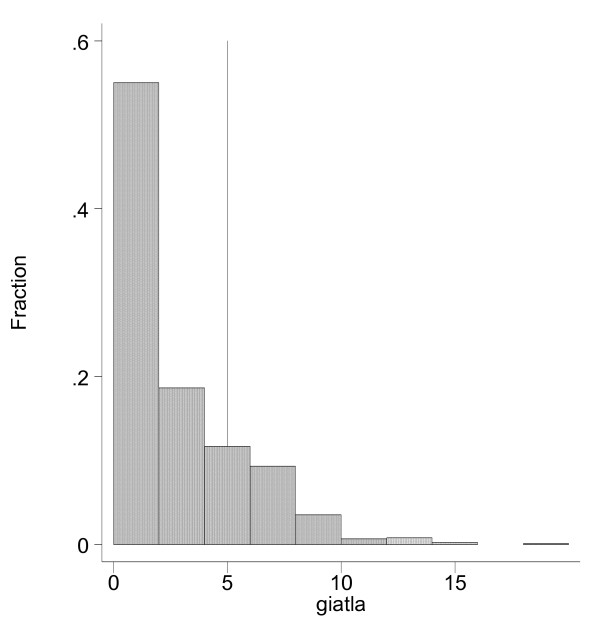
**Distribution of Smokers by Price of Cigarettes**. Source: Author's calculation from VLSS 1998

We compare demographic differences between consumers of high price and low price cigarettes. We categorized by region, by urban and rural status, by occupational type, by educational level (illiterate or only primary education, lower secondary education, and above), and by income. The territory of Vietnam is divided to 7 regions. These regions include both urban and rural areas. Region 1 is the Northern Mountain and Highlands; region 2 is the Red River Delta; region 3 is the North Central Coast; region 4 is the South Central Coast; region 5 is the Central Highlands; region 6 is the Southeast, and region 7 is the Mekong Delta. These 7 regions were used in the regression analysis while controlling for urban status of an individual. In addition to this geographical division, another government classification divides the country into 10 regions. Figure 2 is based on this classification. These 10 regions include the seven regions from the first classification, but cover only the rural population if these 7 regions. The additional 3 regions represent small, medium and large cities. In Figure 2 region 1 represents big cities, region 2 represents middle size towns, region 3 includes small towns in Vietnam, and regions 4–10 represent rural areas in 7 regions used in the regression analysis.

The per capita income is calculated by dividing the total annual household income by the household size. Each individual is assigned to one of five quintiles according to his/her income.

Finally, using expenditures data from the 1998 VLSS, we compared expenditures on tobacco to other items such as education, health care, food, and with the total expenditure of households. We distinguish these comparisons by region, urban-rural and by income. The relative proportion of tobacco spending is important, since if the proportion is high, raising tobacco taxes will have more influence on the household budget.[33]

### Price elasticity estimates

To estimate demand elasticities using cross-sectional data,[34] we employ a method that distinguished the decision to smoke from the quantity smoked. The first equation explains smoking status (indicated by a dummy variable) using the sample of all individuals, and corresponds to a smoking participation equation; the second equation is confined to smokers and uses the logarithm of the number of cigarettes smoked per smoker as the dependent variable, and corresponds to the quantity smoked per smoker or conditional demand equation.

The smoking status model is estimated using the linear probability model (LPM) defined as:

*D*_*smo*ker _= *β*_0 _+ *β*_1 _ln *p*_*Cig *_+ *β*_2 _ln *p*_*Pipe *_+ *β*_3 _ln      (1)

In which:

*D*_*Smoker *_= dummy variable of smoking status (smoker = 1, other while = 0)

*p*_*Cig *_= price of cigarettes at the commune level

*p*_*Pipe *_= price of pipe tobacco

*y *= annual per capita income

*z*_*k *_= variables relate to individual, household, geographic and commune characteristics.

*β*_0 _= constant parameter

*β*_1 _= percentage point change in the possibility of some one being smoker as price of cigarette change 1%

*β*_2 _= percentage point change in the possibility of some one being smoker as price of pipe tobacco change 1%

*β*_3 _= percentage point change in the possibility of some one being smoker as disposable income change 1%

*β*_*k *_Parameter indicating the relationship between individual characteristic and taste, household characteristics, geographic and commune characteristics and the possibility of being a smoker.

The equation design is used to estimate the probability of an individual's smoking status and quantity smoked as a function of cigarettes price while controlling for other variables. The price coefficient indicates the probability of smoking due to variations in cigarette prices. We use communal prices in the models. Since communal prices are provided for 555 and VINATABA brands, we estimated the models with the average price of these two brands, as well as using the prices of just VINATABA or 555. Out of a total of 187 communes, 50 are missing information on the price of VINATABA or 555 and are dropped when using average price. The price variables are converted into logarithmic terms.

The second independent variable is expenditures on pipe tobacco, as a proxy for its price. Pipe tobacco is used by 30.8% of smokers (National Health Survey 2002) and is a potential substitute for cigarettes. As the price of cigarettes increase, smokers may switch to the cheaper pipe tobacco. Since neither the commune nor the household survey collected the price of pipe tobacco or the amount of pipe tobacco consumed, we use information on household spending on pipe tobacco in place of the price of pipe tobacco. Due to potential endogeneity problems (when cigarette prices are high and smoking is reduced, pipe smoking can be expected to increase), we consider equations with and without the pipe variable.

Explanatory variables also include per capita income, characteristics of the individual such as sex, age, education, work experience, occupation; characteristics of the household such as household size and the sex, age, education, main occupation of household head; and commune and geographic characteristics^2^. The commune and geographic variables include urban status, region, and the ability to access employment, markets, transportation. Employment opportunities are captured by the existence of factories or traditional occupations in the commune, access to information is represented by the existence of a loudspeaker system, and access to markets is captured by the presence of regular markets. We used the logarithm of income. Because some rural households have negative incomes (greater investments into production than sales of final goods), we increased all household incomes by 200 thousand VND to make them all positive^3^. Additional File 1 summarizes the variables used in the models.

The conditional quantity model with only smokers is a double-log model[35] with logarithm of number of cigarettes smoked by a smoker as the dependent variable, or:

ln  = *β*_0 _+ *β*_1 _ln *p*_*Cig *_+ *β*_2 _ln *p*_*Pipe *_+ *β*_3 _ln      (2)

Where

ln  = logarithm of quantity of cigarette being consumed by smoker

Other variables are the same as in model (1)

To determine how price responses vary by income, we estimate separate equations by income group. Because income tends to be an unstable measure of overall average living standards of a household, we distinguish by quintiles to examine the low price and high price demand groups. To measure price elasticities of the overall population, the low income group, and the high income group, we estimate one equation for the entire population, one with the two low income quintiles and one with the two high income quintiles, respectively.

The equations were adjusted for clustering using the STATA software package.

### Consumption and tax revenue estimation procedure

Using the information on price elasticities, we estimate the change in consumption and government tax revenue, assuming a uniform tax level of 65%. Because the highest tax rate currently imposed is 65% for filtered cigarette brands produced using imported materials, the price of this category is left unchanged and we only estimate the changes in consumption and tax revenues of unfiltered cigarette and filtered domestic material cigarette categories only. We begin with prices and quantities from the Ministry of Industry and Ministry of Finance.[36]

Currently, taxes are imposed on the wholesale price – the price set by producers. Ignoring any mark-up at the retail level (for simplicity), the price paid by consumer is:

     for tax rate *t*_*0 *_   (3)

     for tax rate *t*_*1 *_     (4)

where:

*t*_*0 *_= original tax rate (25% or 45%)

*t*_*1 *_= increasing the current tax rate to a uniform tax rate (65%)

 = whole sale price by tobacco company while imposing tax rate *t*_*0*_

 = whole sale price by tobacco company while imposing tax rate *t*_*1*_

 = retail price paid by consumer with tax rate *t*_*0*_

 = retail price paid by consumer with tax rate *t*_*1*_

After imposing a higher tax, the total quantity consumed falls, which induces a fall in production. A reduction in output may raise the cost of production which may induce a further increase in the wholesale price (e.g., if there are significant economies of scale in the production of cigarettes), so that  > . However, we assume that  ≈ , because the Vietnamese government already limits the output of cigarette production,[37] so that production may not initially decline with a reduction in consumption. In addition, the reduction in consumption will have little effect on open trade because Vietnam is a price taker in the international tobacco market and can export with a stable price on international markets. We also assume no change in mark-ups at the retail level.

For a percentage point change in tax rate of (Δ_*t*_) = t_1 _– t_0 _and with  ≈ , the percent change in the price as:

*% change in retail price *= Δ_t _P_0_^p^/P_0_^c ^     (5)

As price increases, consumption decreases by an amount that depends on the change in the price and the price elasticity of demand. To estimate the reduction in the quantity consumed (*Q*), we use the price elasticity of demand, , is:



In our model, the estimate of the overall price elasticity is the sum of the estimates of participation and conditional elasticities:



where  is overall elasticity;  is participation elasticity; and  is conditional elasticity.

From equation (6) we obtain:



The magnitude of change in the total cigarette consumption is calculated using the initial quantities of cigarette consumed, the percentage change in prices (due to the change in tax rates) and the overall price elasticity estimates as indicated in (7).

Using equation (7) we obtain the change in consumption as:



Initial government revenue is calculated for each cigarette type as:



Using the estimated  and Δ*P *from (3), we derive Δ*Q *from (7), from which we can calculate *Q*_1_=*Q*_0_-Δ*Q*. With *Q*_1_, we estimate the new government revenue () for each cigarette type as:



The absolute change in the tax revenue is simply .

## Results

### Tobacco consumption patterns

Table 1 describes the smoking prevalence and the smoking intensity in the whole sample and among the 5 income quintiles. The smoking prevalence as well as the smoking intensity are highest among the poorest part of the population. The prevalence of those who only smoke cigarettes is lower among the lowest quintiles, but a higher proportion smoke both cigarettes and pipes (about 5% among the two lowest quintiles and about 3% among the two highest quintiles, not shown).

**Table 1:** Male smoking prevalence and smoking intensity among 5 income quintiles in Vietnam (age 15+; based on VLSS 1998)

**Quintile**	Smoking prevalence (any tobacco use)		Cigarette-only smoking prevalence		Cigarettes/day	
Poor	58.46	56.32	30.02	31.10	12.36	11.90
Near poor	54.19		32.12		11.49	
Middle	52.60		34.94		11.44	
Upper middle	47.70	45.36	36.34	37.20	10.62	10.40
High income	43.02		38.06		10.25	

Whole sample	50.76		34.60		11.11	

The distribution of smokers according to their purchasing price is presented in Figure 2. The dividing point between the low price and the rest is a price of 5000 VND. Based on the Vietnam Living Standards Survey 1997–1998, we found that under-5000 VND per pack smokers accounts for 78% of the market while the over-5000 VND-pack smokers accounts for the rest – 22%. Thus, Figure 2 reveals that the majority of smokers in Vietnam consume low priced cigarettes.

### Characteristics of low-priced cigarette smokers

**Figure 3:**
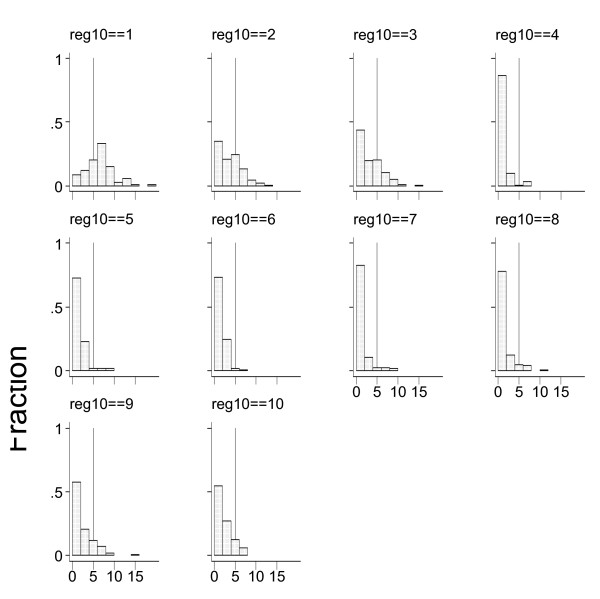
**Distribution of Smokers by Price of Cigarettes and Region**. Source: Author's calculation from VLSS 1998 Notes: Horizontal bar shows price of cigarette (000' VND) Vertical bar shows proportional distribution of cigarette smokers

The prevalence of low-priced cigarette smokers varies with geographic regions, occupations, education levels and household sizes. Figure 3 shows the distribution of smokers by cigarette prices and by large, medium and small urban areas (region 1, 2 and 3, respectively) as well as for seven rural regions from North to South. One-third of smokers in large cities smoke low-priced cigarettes. The proportion increases as one moves to medium then small cities and finally is highest in rural areas.

The low-priced cigarettes are particularly popular among households whose heads are agricultural workers, which includes about 60% of low-priced smokers. Further, about 11% of low-priced smokers live in households whose heads are jobless. Thus, the majority of low-priced cigarette smokers live in rural areas, where tobacco control activities are quite limited.[38] In analyzing education level, low-priced cigarette smokers mostly live in households whose heads have low levels of education: 69% live in households where the head is illiterate or has only a primary education, and 16% live in households where the head has a lower secondary school education.[39]

### Tobacco expenditures by smokers

Table 2 compares tobacco spending with spending on other items such as education, health care, food and with the total expenditure of households. Rural households have a higher ratio than urban households of tobacco spending to education spending (the respective figures are 71% and 42%). Rural households' other proportions including tobacco spending to health care spending, tobacco spending to food spending, and tobacco spending to total expenditure are also higher than urban households. Tobacco spending is also higher in southern regions.

**Table 2:** The comparison between tobacco spending with education, health care, food spending and total expenditure of households by quintile (%)

	Tobacco spending/education spending	Tobacco spending/healthcare spending	Tobacco spending/food spending	Tobacco spending/total expenditure
Total	62.47	56.23	6.36	3.48
Urban-rural				
Rural	71.36	56.91	6.43	3.67
Urban	42.31	53.79	6.11	2.92
Large city	29.70	31.77	4.95	2.15
Small city and town	41.38	54.25	5.93	2.79
Small town	51.01	72.09	6.83	3.46
Region				
North Mountain and Midland	63.44	63.52	4.18	2.60
Red River Delta	59.98	57.76	5.04	2.87
North Central Coast	67.87	62.19	6.40	3.69
South Central Coast	83.96	67.19	6.98	4.06
Central Highlands	81.70	75.65	6.22	3.75
Southeast	53.70	47.60	6.82	3.61
Mekong Delta	83.77	52.47	7.48	4.19
Income				
Very low Income	150.69	97.11	7.83	5.29
Low	108.17	86.99	7.31	4.58
Middle	94.21	67.00	7.40	4.30
Mid-High	68.10	72.69	7.71	4.06
High	46.35	68.92	8.83	3.60

Source: The Vietnam Living Standards Survey 1997–1998

While it might be expected that low income households spend a larger part of their income on basic needs such as clothing, education, health care, housing, transport, and a smaller part on tobacco, we found that the tobacco spending of low income households represents a larger proportion of their expenditure than for higher income households. Low income households' tobacco spending is equal to one-and-a-half times their educational spending and is equivalent to health care spending. By contrast, tobacco expenditures for higher income households are 46% and 69%, of educational and health expenditures, respectively. The percentages of low income households and higher income households' tobacco spending are 5.3% and 3.6%, respectively.

### Estimating the price elasticity of tobacco demand

#### The smoking participation model

Table 3 presents the results of model estimated with VINATABA price. The participation model was estimated with three alternative price variables: VINATABA, 555 brand and average price. However, the price coefficient for 555 brand was often not significant at the 10% level and are not presented in this paper, but can be obtained upon request. The coefficients of log (VINATABA) price are significant at the 10% level or lower. Since the price elasticity of participation is equal to the coefficient on the logarithm of the price variable divided by the smoking prevalence among men (56% Vietnam National Health Survey 2002), we estimate that the price elasticity of male participation in smoking is  =-0.94 for whole male population,  =-1.16 for the two low quintiles and  = -0.75 for the two high quintiles.

**Table 3:** Result from equation explaining the decision to participate to smoking, dependent variable is smoking participation (yes = 1, no = 0)

Independent variables	Overall		Two low quintiles		Two high quintiles	
	
	Coef.	P > | t	Coef.	P > | t	Coef.	P > | t
Logarithm of VINATABA price	-0.529	0.048	-0.648	0.086	-0.420	0.094
Expenditures on pipe	-0.011	0.015	-0.011	0.016	-0.010	0.023
Year of education	-0.176	0.231	-0.167	0.255	-0.163	0.264
Age	0.500	0.070	0.442	0.109	0.429	0.119
Year of experience	-0.495	0.080	-0.435	0.123	-0.424	0.132
Household size	0.961	0.000	0.965	0.000	0.950	0.000
Logarithm of per capita income	2.415	0.003	2.380	0.003	2.421	0.003
Red river delta (yes = 1)	4.178	0.000	4.117	0.000	4.088	0.000
North central coastal (yes = 1)	1.588	0.580	1.634	0.568	1.689	0.554
South central coastal (yes = 1)	6.972	0.001	6.076	0.004	6.165	0.003
South-east region (yes = 1)	9.618	0.000	9.615	0.000	9.662	0.000
Mekong delta (yes = 1)	11.051	0.000	11.019	0.000	10.137	0.000
Urban (yes = 1)	9.104	0.000	9.124	0.000	9.076	0.000
Existence of regular market (yes = 1)	0.290	0.798	0.279	0.805	0.284	0.801
Opportunity to find job (yes = 1)	-1.081	0.341	-0.994	0.380	-0.988	0.381
Accessibility of car to commune (yes = 1)	-0.929	0.720	-0.849	0.742	-0.864	0.737
_constant	-39.358	0.000	-38.235	0.000	-38.182	0.000
Number of observation	5202		2412		2023	
R-squared	0.239		0.198		0.197	

Source: Author estimation from VLSS 1997–1998.

The results also indicate that coefficients of smoking expenditures on pipe tobacco, household size, income and region are statistically significant, but the variables for access to markets, jobs and transportation are not significant. The coefficients on income, 2.380 for high income households and 2.421 for low income household, are high and significant in both models. As the income of Vietnamese rural households increases, smoking prevalence is on the rise in those rural areas.[40] However, the magnitude of the income elasticity has to be viewed with a caution since Vietnamese households do not tend to report their true income.

#### The conditional quantity model

The results of this model are presented in Table 4 (Full detail results are presented in Additional File 2). We estimated a regression with all smokers, then for the two lowest quintiles, for the two highest quintiles and then for the middle quintile. The models were separately estimated with the VINATABA and 555 communal prices and average communal price. The price elasticity of the middle quintile is not significant and not presented in the table. If we take the average price elasticity of the equations with the prices entered separately (both were significant at the 10% level), the conditional price elasticity of the overall population is  = -0.50, for the two low quintiles is  = -0.59 and for the two high quintiles, it is  = -0.40.

**Table 4:** Estimated price elasticity of conditional demand based on different prices

	Overall	Two low quintiles	Two high quintiles
Communal VINATABA price	-0.469*	-0.613*	-0.422*
Communal 555 price	-0.538**	-0.574*	-0.372**
Communal average prices	-0.497	-0.845	-0.348

Average price elasticity of conditional demand using estimates for 555 and VINATABA (the significant estimtes)	-0.504*	-0.594*	-0.397*

Source: Author's estimation from VLSS1998

Note: Commune average price is average of 555 & VINATABA prices

** is below 5% of significant level,

* is below 10% of significant level.

#### The overall price elasticity of cigarette demand

The overall price elasticity of cigarette demand was calculated by adding the price elasticity of conditional demand and the price elasticity of smoking intensity (quantities) for the respective income groups. We used estimates from the model using VINATABA price. We found that the overall price elasticity for the whole population is  = -1.41, for the two low quintiles is  = -1.77 and for the two high quintiles, it is  = -1.17.

### The effect of imposing a uniform tobacco tax on consumption and tax revenues

Tables 5 and 6, respectively, show the consumption and tax revenues under the current tax regime and with an increase in taxes for the lower taxed categories to a uniform 65% tax. From Table 7 we can see that by imposing a uniform tobacco tax rate of 65% (compared to the former 25% rate), the price of low-price cigarettes will increase 32% while the price of the second category (formerly 45%) increases by 16%. Government revenues from the tax increase 11.5% or 127.5 billion VND ($8.5 million), of which 14.1 billion VND are from the former 25% category and 113.39 billion VND from the former 45% category. Most importantly, total cigarette consumption falls 25.3% for both categories, with consumption of the first category falling 46.1% and the second category falling 23%.

**Table 5:** Base Consumption, price and revenue with current tax

	Quantity consumed (Million packs)	Retail price (1000 VND)	Tax rate based on whole sale price	Tobacco tax revenue (Billion VND)
Non-filtered cigarettes	175.4	1.00	0.25	35.1
Filtered cigarettes produced using domestic materials	1578.2	2.20	0.45	1077.5

Total	1753.5			1112.6

*Source*: Ministry of Industry and Ministry of Finance, 2003^49^.

**Table 6:** Consumption, price and revenue after imposing a uniform high tobacco tax of 65%

	Quantity to consume (Million packs)	Price after tax (1000 VND)	Tax rate	Tobacco tax revenue (Billion VND)
Non-filtered cigarettes	94.51	1.32	0.65	49.2
Filtered cigarettes produced using domestic materials	1215.2	2.55	0.65	1190.9

Total	1309.7			1240.0

*Source*: Author's Estimation

**Table 7:** Change in price, consumption & government tobacco tax revenue from imposing a uniform high tobacco tax rate of 65%

	Change in price (%)	Change in Tobacco Consumption		Change in Tobacco Tax Revenue^(2)^	
		Quantity (Million packs)	%	Value (Billion VND)	%

Non-filtered cigarettes	32%	-80.8	-46.1	14.1	40.2
		
Filtered cigarettes produced using domestic materials	16%	-363.0	-23.0	113.4	10.5

Total		-443.8	-25.3	127.5	11.5

*Source*: Author's estimation

## Conclusion

Currently, Vietnam imposes three different tax rates on tobacco according to the source of raw materials and whether filtered or non-filtered. The large discrepancies among these rates lead to large disparities among cigarette prices and wider availability of low-priced cigarettes in the market, which makes it easier for youth and the poor to get access to tobacco.

The evidence presented here indicates that most smokers smoke low priced cigarettes. Most consumers of low priced cigarettes are poor and live in rural areas or small towns; they tend to be employed in the agricultural sector. Low income households' tobacco spending is equal to one-and-a-half times their educational spending and is equivalent to health care spending. Higher income smokers also spend a large share of their income on tobacco, but the poor bear the largest relative economic burden. Because of their high smoking rates, the poor are also more likely to have higher risks of tobacco-related diseases such as cancer, heart and circulatory diseases, and emphysema.[41] In addition, because these diseases can appear as early as age 40 (along with other smoking related medical conditions), the higher disease incidence is likely to further increase the economic burden by increasing the likelihood of not being able to continue to work and earn income. Thus, social goals of reducing poverty would dictate reducing tobacco spending by the poor.

With sufficient responsiveness, taxes may encourage reductions in spending. Unlike previous most previous demand studies, this study considers how price responsiveness varies by income. The price elasticity of male participation in smoking is  = -0.94 for the whole population. However, demand for the low income group ( = -1.16) is more elastic than for the high income group ( = -0.75), as might be expected due to their more limited budgets. The price elasticity estimates are high compared to evidence from other developing countries. Even though these estimates wary substantially from a country to country, the majority of estimates for the total price elasticity centers around  = -0.8.[42] However, these estimates do not take into account substitution to other tobacco products. Our results are quite consistent with Laxminarayan and Deolalikar (2004)[43] who estimated a price elasticity of smoking initiation of -1.18 for Vietnam, and generally consistent with other studies for Southeast Asia.

The high participation elasticity indicates that raising the price to low income consumers will lead to substantial reductions in the number of cigarette smokers, enough to reduce overall expenditures on tobacco by the poor. Further reductions will occur through reductions in the quantity smoked by those continuing to smoke. As the price of cigarettes increases 10%, the quantity demanded of low income smokers that continue to smoke falls 5.9% while the quantity demanded of high income smokers falls by only 4.0%. The overall quantity demanded among smokers falls 5.0%. Thus, of those who continue to smoke, there will only be modest increases in tobacco expenditures, with a smaller increase among the poor compared to the higher income groups.

Imposing a uniform tax of 65% on tobacco will result in a 32% increase in the prices of low-priced cigarettes and a 16% increase for the domestic filtered category. Applying the overall price elasticity of cigarette demand of -1.44, we predict a decrease of tobacco consumption of about 25%, and an increase of more than 11% ($8.5 mil) in the tobacco tax revenue of the government. A study[22] using a price elasticity of -0.53 predicted a 9.3% decrease in consumption and 33.5% (or $24.8 mil) increase in government revenue.

The results indicate that tax revenues are likely to increase as tax rates are increased for domestic unfiltered and domestic filtered to the rate for foreign filtered cigarettes. Although quantities consumed fall, the increase in tax rate more than compensates. Thus, government revenues increase despite the large reductions in consumption, suggesting that government could benefit from the moving to a uniform tax.

While we used a linear probability model for ease of interpretation and use, the error term is heteroskedastic and is not normally distributed, the predicted values are not constrained to be between 0 and 1. Nevertheless, the results reported here on price effects are consistent with the theory of cigarette demand and empirical evidence from other countries.[44] Still, due to data limitations and simplifying assumptions, the results should be regarded with caution.

First, income tends to be reported incorrectly in Vietnam. Surveyed subjects tend to declare lower income because high income is often regarded as being linked to illegal activities and corruption. However, despite measurement error, the results indicate high sensitivity of cigarette demand to income. As the income is expected to grow in the near future in Vietnam, there is a danger that smoking prevalence will grow if no tobacco control measures are taken.

A second limitation is that the potential substitution into pipe smoking was taken into account by using expenditures on pipe tobacco, instead of price, creating potential endogeneity problems. When we omitted the pipe variable, we obtained consistent results, with slightly lower coefficients and levels of significance on some of the price variables. Nevertheless, in Vietnam, pipe tobacco is a likely an important substitute for cigarettes, especially among the poor. With an increase in the consumption of pipe tobacco, the price of pipe tobacco should increase, reducing some of the pipe use. However, any substitution into pipe use may still be preferable to smoking if pipe smoking is less expensive and imposes less economic burden on the poor and if pipe use has less harmful effects on health. Further work is needed on the role of substitution into pipe use and the health effects of pipe use relative to that of smoking.

A third limitation is that the price data is measured with error. This is, in part, because we were able to only include the price of medium and high price standard brands. We did, however, obtain relatively similar results with the various combinations of the measures, although the results were not always significant, as might be expected with measurement error. We also estimated equations which considered prices based on the expenditures on tobacco and quantity smoked (both undoubtedly measured with error), and obtained roughly consistent results. In computing the change in tax revenues, we did not consider substitution between cigarette types. As the price of the cheaper, non-filtered domestic increases, there may be substitution toward the filtered domestic brand, and, as the filtered domestic cigarettes increases, there may be more substitution towards the filtered cigarettes with foreign tobacco. However, these effects would tend to increase tax revenues as more cigarettes are purchased of the more expensive brands. For these reasons, we may have underestimated the increase in tax revenues, but overestimated the effect on consumption.

We do not consider smuggling or domestic tobacco growing. Imposing a uniform tax on cigarettes will increase the domestic market price of cigarettes and may widen the gap between domestic and the prices of cigarettes in neighboring nations providing more motivation for smuggling. In addition, higher market prices of cigarettes may encourage an increase in domestic tobacco growing. These changes in the tobacco market may reduce the tax revenues. However, a review of the literature[45] indicates that smuggling depends on factors other than the gap between domestic and international prices of cigarettes. These factors include the strength and extent of law enforcement, activities of market control forces, and the living standards of poor people in border areas (poor, jobless people near some border entrance points tend to work for smugglers to move cigarettes into Vietnam). The Government of Vietnam currently pays considerable attention to smuggling in order to protect the domestic market. Nevertheless, the smuggling issue and the issue of domestic tobacco growing as a response to cigarette price changes merit further study.

We extrapolate future tax revenues from a single price elasticity estimate. In addition to smuggling, our estimates will depend on income, population and other tobacco control policies, among other factors. Rising income and population will increase tax revenues, but other tobacco control measures will reduce demand for cigarettes and, hence, tend to reduce revenues.

We focus on male participation. Tobacco use by women is much lower, with 1.8% of females smoking in 2002 and 50% of female smokers smoking pipes. Since smoking is prohibited by some parents, and may be considered improper for women (Do Hong Ngoc 1995[46]), it is likely that there is some underreporting of cigarette and tobacco use among women, as well as youth. Nevertheless, it will be important to monitor female smoking in future years since these social norms may change with the rapid economic development experienced by Vietnam. The lack of awareness of the health risk associated with smoking may then result in a fast onset of female smoking.[47]

Finally, the optimal tax rate that should be imposed is difficult to determine because it depends on many factors including the costs imposed by smokers on non-smokers. Another potentially important concern is employment in both the tobacco industry and tobacco cultivation. When imposing a high tax, the reduction in consumption may reduce employment in some areas. But the effect is also not clear since the tobacco industry can export both tobacco leaf and cigarettes. The choice of tax rate also depends on social values such as the protection of children and others from tobacco smoke, and on the purpose of taxation, i.e., to increase tax revenues or to reduce the burden of tobacco related diseases.

Worldwide, only few countries levy different tobacco tax rates on cigarettes. Most of them are former socialist economies in Eastern Europe and the former Soviet Union where this phenomenon was inherited from the planned economy structure. The differential treatment of various cigarette types can be justified neither on economic nor on public health grounds since all cigarettes are harmful to health.

An increase of tobacco taxes can have additional positive impact on public heath if part of tobacco tax revenue is earmarked for anti-smoking activities. Victoria, Australia was the first jurisdiction to establish a health promotion agency funded by tobacco taxes. Many others similar agencies were modeled upon the Victorian Health Promotion Foundation, such as ThaiHealth Foundation in Thailand. Thailand is one of the countries with the greatest success in tobacco control. The tobacco tax increased from 55% in 1992 to 75% in 2001, which is applied as a uniform tax for all tobacco products. Meanwhile the tobacco tax revenue doubled from 15,438 million Baht in 1992 to 31,247 million Baht in 2002 and consumption fell from 2,035 million packs in 1992 to 1,716 million packs in 2002.[48] Thus, other tobacco control activities may further supplement the effect of a tax increase.

In this study, we found that the poor are more responsive to cigarette prices than those with higher incomes. Consequently, a tax increase would lead to relatively higher reduction of cigarette demand among the lowest income group and generally encourage that group to reduce the large economic burden that smoking imposes.

## Note

^1 ^VND is an abbreviation for the local currency in Vietnam called Vietnamese Dong

^2 ^The two poorest regions in Vietnam were used as a benchmark

^3 ^200 thousand VND is the smallest amount that makes income for households positive when added to the reported income.

## Supplementary Material

Additional File 1 Contains the list of variables in the used in the logit and regression models. Click here for file

Additional File 2 Contains the results from the Conditional Quantity Regression Analysis. Click here for file
