# Supplementary material for: The effect of imposing a higher, uniform tobacco tax in Vietnam
Source: Health Res Policy Syst. 2006 Jun 26;4:6. doi: 10.1186/1478-4505-4-6 (PMC1557504; doi:10.1186/1478-4505-4-6)
Supplement: Additional File 1 — Contains the list of variables in the used in the logit and regression models. [file 1478-4505-4-6-S1.doc]

**Additional File 1**

| **Appendix Table 1: Constructed variables for running regressions** | |
| --- | --- |
| Variable | Explanation |
| ***Variables related to individual characteristics*** | |
| edu | Education by level attained |
| eduyear | education by years of school |
| age | age |
| exper | work experience |
| child | indicates a child |
| exper2 | experience square |
| sex | sex |
| smokcig | cigarette smoker |
| num_cig | number of cigarettes consumed by smoker |
| moneycig | spending on cigarettes by smoker |
| pipe | pipe smoker |
| smoker | smoker who either smoked cigarettes or pipe tobacco or both |
| moneypip | spending on pipe tobacco |
| lnumber | logarithm of number of cigarettes consumed by smoker |
| mainfarm | main occupation is farming |
| mainoccu | main occupation of individual |
| mainsect | main sector that individual works for |
| whour | number of working hours by individual |
| wpeop | dummy variable for working people |
| dpend | dummy variable for dependents |
| ***Variable related to household characteristics*** | |
| headedu | education of household head by level attained |
| heduyear | education of head of household by years of school |
| headage | age of head of household |
| headsex | sex of head of household |
| hchild | number of children |
| avexper | average experience of other working member (not including the head) |
| avedu | average educarion level of other working member (not including the head) |
| avexper2 | average work experience of other working members (not including the head) squared |
| cluster | cluster |
| hhsmoke | number of smokers in the household |
| hhnumcig | quantity of cigarettes consumed by household |
| hmonecig | spending of household on cigarettes |
| hmonpipe | spending of household on pipe tobacco |
| hwhour | number of working hours of household members |
| headhour | number of working hours of head of household |
| hwpeop | number of working people |
| hdpend | number of dependents |
| headreti | dummy variable if head of household is retired |
| headself | head is self employment |
| headoccu | occupation of head of household |
| headsect | the economic sector where the household head works |
| quint98 | quintile |
| lnpcearn | logarithm of annual per capita income |
| lhhdieu | logarithm of number of cigarette consumed by household |
| quint2 | dummy variable of quintile 2 |
| quint3 | dummy variable of quintile 3 |
| quint4 | dummy variable of quintile 4 |
| quint5 | dummy variable of quintile 5 |
| hhsize | household size |
| wt | individual weight |
| hhsizewt | household weight |
| ***Variable relate to commune characteristics*** | |
| commune | commune |
| lpvina | logarithm of communal price of VINATABA brand |
| lpvina | logarithm of communal price of 555 brand |
| lvina555 | logarithm of communal average price |
| road | car can access the commune all year round |
| market | existence of regular market in the commune |
| findjob | existence of factories or traditional occupation |
| urban | dummy variable urban=1 |
| reg7 | region by seven regions |
| reg10 | region by ten regions |
| region1 | dummy variable of region 1 |
| region2 | dummy variable of region 2 |
| region3 | dummy variable of region 3 |
| region4 | dummy variable of region 4 |
| region5 | dummy variable of region 5 |
| region6 | dummy variable of region 6 |
| region7 | dummy variable of region 7 |
