# Supplementary material for: The effect of imposing a higher, uniform tobacco tax in Vietnam
Source: Health Res Policy Syst. 2006 Jun 26;4:6. doi: 10.1186/1478-4505-4-6 (PMC1557504; doi:10.1186/1478-4505-4-6)
Supplement: Additional File 2 — Contains the results from the Conditional Quantity Regression Analysis. [file 1478-4505-4-6-S2.doc]

**Additional File 2**

**Table 2: Result with dependent variable of logarithm of number cigarette consumed**

First variable is logarithm of Vinataba commune price

First variable is logar

ithm of 555 commune price

First variable is logarithm of average Vinataba & 555 commune prices

Variable

Overall

Two low quintiles

Two high quintiles

Overall

Two low quintiles

Two high quintiles

Overall

Two low quintiles

Two high quintiles

Coef.

P>|t

Coef.

P>|t

Coef.

P>|t

Coef.

P>|t

Coef.

P>|t

Coef.

P>|t

Coef.

P>|t

Coef.

P>|t

Coef.

First variable

-0.469

0.085

-0.613

0.095

-0.422

0.127

-0.538

0.037

-0.574

0.020

-0.372

0.026

-0.497

0.139

-0.845

0.291

-0.348

hmoneypi

0.000

0.868

0.000

0.943

0.000

0.877

0.000

0.299

-0.001

0.068

-0.001

0.101

0.000

0.737

0.000

0.001

-0.001

eduyear

-0.014

0.621

-0.019

0.558

-0.027

0.453

0.001

0.892

0.002

0.820

0.001

0.917

-0.010

0.706

-0.018

0.032

-0.011

age

0.059

0.119

0.069

0.117

0.082

0.090

-0.002

0.891

-0.005

0.722

-0.002

0.874

0.054

0.147

0.068

0.044

0.049

exper

-0.061

0.112

-0.072

0.107

-0.084

0.084

0.006

0.717

0.009

0.544

0.006

0.711

-0.057

0.138

-0.071

0.044

-0.052

sex

-0.438

0.139

-0.168

0.657

-0.377

0.412

0.026

0.836

0.058

0.643

0.026

0.836

-0.468

0.114

-0.186

0.377

-0.408

wage

0.000

0.267

0.000

0.031

0.000

0.024

0.000

0.890

0.000

0.886

0.000

0.962

0.000

0.259

0.000

0.000

0.000

hhsize

0.041

0.006

0.059

0.021

0.062

0.022

0.017

0.029

0.015

0.064

0.016

0.046

0.039

0.008

0.055

0.025

0.041

lnpcearn

0.143

0.018

0.165

0.083

0.124

0.221

0.024

0.087

0.020

0.136

0.017

0.086

0.131

0.047

0.161

0.094

0.142

region2

0.051

0.895

0.084

0.832

0.007

0.186
